# Understanding self-reported importance of religion/spirituality in a North American sample of individuals at risk for familial depression: A principal component analysis

**DOI:** 10.1371/journal.pone.0224141

**Published:** 2019-10-18

**Authors:** Connie Svob, Lidia Y. X. Wong, Marc J. Gameroff, Priya J. Wickramaratne, Myrna M. Weissman, Jürgen Kayser

**Affiliations:** 1 Department of Psychiatry, College of Physicians and Surgeons, Columbia University, New York, New York, United States of America; 2 New York State Psychiatric Institute, New York, New York, United States of America; University of Auckland, NEW ZEALAND

## Abstract

Several studies have shown protective effects between health outcomes and subjective reports of religious/spiritual (R/S) importance, as measured by a single self-report item. In a 3-generation study of individuals at high or low familial risk for depression, R/S importance was found to be protective against depression, as indicated by clinical and neurobiological outcomes. The psychological components underlying these protective effects, however, remain little understood. Hence, to clarify the meaning of answering the R/S importance item, we employed a comprehensive set of validated scales assessing religious beliefs and experiences and exploratory factor analysis to uncover latent R/S constructs that strongly and independently correlated with the single-item measure of R/S importance. A Varimax-rotated principal component analysis (PCA) resulted in a 23-factor solution (Eigenvalue > 1; 71.5% explained variance) with 8 factors that, respectively, accounted for at least 3% of the total variance. The first factor (15.8%) was directly related to the R/S importance item (*r* = .819), as well as personal relationship with the Divine, forgiveness by God, religious activities, and religious coping, while precluding gratitude, altruism, and social support, among other survey subscales. The corresponding factor scores were greater in older individuals and those at low familial risk. Moreover, Spearman rank-order correlations between the R/S importance item and other subscales revealed relative consistency across generations and risk groups. Taken together, the single R/S importance item constituted a robust measure of what may be generally conceived of as “religious importance,” ranking highest among a diverse latent factor structure of R/S. As this suggests adequate single-item construct validity, it may be adequate for use in health studies lacking the resources for more extensive measures. Nonetheless, given that this single item accounted for only a small fraction of the total survey variance, results based on the item should be interpreted and applied with caution.

## Introduction

There has been growing interest in the role of religiosity/spirituality (R/S) in health research [1], with findings generally focusing on three broad R/S domains based on single-item measures, namely, R/S importance, service attendance, and religious affiliation. R/S importance (i.e., *How important to you is religion or spirituality*?) is often associated with protective effects—presumably independently of religious service attendance and religious affiliation, which have also proven beneficial [2–7]–and this has been particularly true in our studies [8–16]. As such, we were motivated to investigate what underlies the largely subjective and complex construct of R/S importance.

As reviewed by Koenig [1], approximately 80% of research on R/S and health focuses on mental health. Previous research by our team has investigated extensively the ways R/S importance, service attendance, and denomination impact families at-risk for major depressive disorder. In most instances, we found that R/S importance was the only item associated with many of the health-related outcomes. For example, we found high self-report ratings of R/S importance to be protective against recurrence of depression [11] and childhood suicidal behaviors [12]. We have also observed protective effects of R/S importance in biomarkers of high-risk as compared to low-risk families, including decreased default mode network connectivity [13], thicker cortices [8–9], and greater posterior EEG alpha [15–16]. Moreover, these effects were transmitted across generations for both depression [10] and suicidal behavior [14], and extended to correlates of genetic markers [17]. Taken together, R/S importance has evidenced a role in the resilience of individuals at high risk for depression [18–19], yet the specific mechanisms that underlie its protective effects remain little understood.

There is general consensus among social scientists that a single item is insufficient to describe the depth and complexity of personal religiosity and spirituality [20–30]. To this end, various efforts have been made to (a) differentiate explicitly between religion and spirituality [1,28–29], and (b) to design multidimensional instruments that capture different aspects of religious/spiritual experience [20,28,31]. Importantly, how participants understand or respond to the single R/S Importance item, whose terms are not defined and are, moreover, conflated in the single item, remains unknown.

Efforts to expand measures of this single item have primarily been driven through a top-down process, that is, they have been based on theoretical constructs that hold significance to psychologists and religious scholars. In contrast, McClintock, Lau, and Miller [28] developed a questionnaire to identify common dimensions of spirituality across three diverse cultures (China, India, USA). Their objective was to examine the spiritual constructs that may not be captured by more traditional scales administered to primarily Judeo-Christian populations. Using exploratory factor analysis and cross-validating exploratory structural equation modeling identified five factors: (a) Altruistic Engagement (i.e., altruism), (b) Love, (c) Contemplative Practice (e.g., meditation, yoga), (d) Unifying Interconnectedness (viewing the environment and all living things from a spiritually unifying perspective), and (e) Religious and Spiritual Reflection and Commitment. Using confirmatory factor analysis on a selected subset of their original 34 R/S instruments, McClintock et al. [27] were able to replicate this factor structure in the present sample of individuals at high and low risk for major depressive disorder. Importantly, the R/S Reflection and Commitment factor correlated strongly (*r* = .80) with the single-item of R/S importance, and lower religious/spiritual commitment was associated with previous MDD diagnosis, particularly among high risk individuals. Because the recent McClintock et al. [27] study was motivated by the findings of McClintock et al. [28], it only included about two-thirds of the available R/S variables for this sample (i.e., to maximize variable overlap across these two McClintock et al. studies), and clarifying the meaning of the single-item of R/S importance was not its primary focus.

Therefore, the purpose of the present study was to conduct an in-depth investigation of the single item of R/S importance through a bottom-up, data-driven process, which would reveal the psychological components underlying the R/S importance item. The obvious advantage of a data-driven approach is its bottom-up nature to inform our understanding of the psychological construct of R/S importance, which should, in turn, provide important clarifications for previous and future health-related findings. Hence, the goal of the present study was to better understand what underlies the protective effects observed in prior reports that have found associations with high ratings of R/S importance. To this end, we employed a comprehensive survey to examine the relationship between the single item measure of R/S importance and other validated constructs within the psychology of religion. By doing so we sought to help bridge previous theoretical and empirical findings across the biomedical and social sciences and aid in their interpretation.

## Methods

### Participants

Data were derived from a 3-generation, 35-year longitudinal study of families at high- and low-risk for major depressive disorder (MDD) that also included reports of R/S importance [18–19]. Over the 35 years, data have been collected at Year 0 (baseline) and in subsequent waves at Year 2, 10, 20, 25, 30, and 35. High and low risk was defined by the original proband (1st generation; G1) having a diagnosis of major depressive disorder (MDD), or no psychiatric diagnosis, as related to their 2nd generation (G2) and 3rd generation (G3) offspring. G1 participants were all European Caucasian and predominantly Catholic. In the present study, participants (*N* = 282) were drawn from both risk groups and across all generations, with ages ranging from 18.5 years to 87.4 years. All participants provided written informed consent and all interviews were approved by the Institutional Review Boards at Columbia University and New York State Psychiatric Institute. Data for the present study were collected 35 years into the study (Yr35) and included an extensive survey of R/S measures as detailed below, comprising Likert-scale items that were administered using paper and pencil (*n* = 88) or online (*n* = 194) through a secure, HIPAA-compliant internet application (Qualtrics.com).

### Religiosity/Spirituality variables

Data collection in this ongoing longitudinal study has typically been separated by approximately 5 to 10 year increments since Year 10 (i.e., at Years 0, 2, 10, 20, 25, 30, and 35). Starting at Year 10 and continuing to the present (Year 35), two single items of religious/spiritual items of importance and service attendance have been measured.

R/S importance (R/S IMPORTANCE) was measured by responses to the question *How important to you is religion or spirituality*? on a 4-point Likert scale (i.e., *highly*, *moderately*, *slightly*, or *not at all important*).

Religious service attendance (REL_ATTENDANCE) was determined by responses to the question *How often*, *if at all*, *do you attend church*, *synagogue*, *or other religious or spiritual services*? on a 5-point Likert scale (*Once a week or more*, *About once a month*, *About once or twice a year*, *Less than once a year*, or *Never*).

In addition to measures of R/S importance and attendance at Year 35, we also obtained data for an extensive array of religious and spiritual constructs taken from previously validated, published scales. This survey sought to include a reasonably comprehensive set of religious (facets of organized religion) and spiritual constructs (encompassing the broader spiritual dimension of the human person not dependent on affiliation with formal institutional religion). It drew heavily upon Hill and Pargament’s [32] suggestions for areas of growth concerning religious conceptualization and measurement in health research, as well as McClintock et al.’s [28] global spirituality measures to encompass a diverse array of religious and spiritual constructs. Notably, the present survey included several R/S instruments (i.e., compassion, forgiveness, gratitude, religious community support, religious coping, self-transcendence, social support, volunteering) that were not considered by McClintock et al. [27]. See Table 1 for complete listing (in alphabetical order).

**Table 1:** Religious/Spiritual constructs comprising survey.

R/S Construct	LABEL	Number of Items	Description	Reference
*Altruism*	ALTRUISM	6	Giving of oneself for the good of another.	[33]
*Belief Salience*	BEL_SALIENCE	5	Degree to which religious beliefs influence one’s personal life and God is considered an intimate part of it.	[34]
*Compassion*	COMPASSION	5	Empathy for others.	[31]
*Contemplative Practice*	CONT_PRACT	4	Mind-body practices, such as yoga and meditation.	[28]
*Eco-Awareness*	ECO_AWARE	6	Degree to which communion is sensed with creation and all living things.	[35]
*Forgiveness*	FORGIVE	3	A measure of forgiveness extended to self, others, and God.	[31]
*Gratitude*	GRATITUDE	4	Recognition and appreciation of an inherent good.	[31]
*Intrinsic Religiosity*	INTRINS_REL	3	Degree to which a person is motivated by religious precepts for their own sake, rather than the perceived benefits they receive from being a part of a religious group.	[36]
*Ontological Love*	ONT_LOVE	4	Attitudes toward love.	[37]
*Psychological Love*	PSYC_LOVE	4	Experience of being loved.	[37]
*Religious Community Social Support*	REL_SUPPORT	2	Degree to which a religious community itself lends support to a person, aside from the support found in other parts of one’s life.	[20]
*Religious Coping*	REL_COPING	6	Degree to which a person uses religion to help them cope with life stressors by either framing situations in a positive light or seeking refuge and support in God.	[31]
*Religious Engagement*	REL_ENGAGE	4	Degree to which a person engages in religious activities, such as prayer, reading sacred texts, attending services, participating in religious groups like Bible Studies.	[33]
*Self-Transcendence*	SELF_TRANS	26	Degree to which religion or spirituality elevate a person’s awareness beyond themselves.	[38]
*Social Love*	SOC_LOVE	4	Relational love.	[37]
*Social Support*	SOC_SUPPORT	3	Support derived from friends and family.	[31]
*Spirituality in Nature*	SP_NATURE	7	Sensing a greater spiritual power through nature.	[28]
*Universality*	UNIVERSALITY	9	Awareness of interconnection between oneself, others, and all of life.	[39]
*Volunteering*	VOLUNTEER	1	Freely giving of one’s time to those in need, whether religious or non-religious.	[31]

### Statistical analyses

Given the research objective, a multi-pronged statistical approach was adopted. First, Spearman rank order correlations were run between R/S importance and the survey items at Yr35 across high and low risk groups, as well generations 2 and 3. Generation 1 (G1) was precluded in the refined analyses as the G1 probands defined risk status for all following generations. Survey items were then ordered by their degree of association with the R/S importance item at Yr35. Second, all survey items (excluding items related to follow-up questions) were submitted to exploratory factor analysis using SPSS 24.0 (SPSS Inc., 2011). Beforehand, missing values were imputed in the statistical platform *R* on a variable-by-variable basis using the package MICE (Multivariate Imputation via Chained Equations) [40]. After excluding follow-up items on Religious Coping (REL_COPING_1 to REL_COPING_6) and Religious Support (REL_SUPPORT_1 and REL_SUPPORT_2), 101 variables were used for imputation of 0.54% of data assumed to be missing at random. Five imputed datasets were created using predictive mean matching (50 iterations) and then combined to an average dataset with no missing values. The final imputed data set comprising 101 variables was submitted to principal component analyses (PCA) using a correlation association matrix, followed by an orthogonal rotation (Varimax) [41] to maximize item variance and simplify interpretability of the latent factors. As there was no a priori assumption about the factor structure, that is, to what degree factors are correlated, this process was repeated using an oblique rotation (Promax) [42]. Factor extraction was limited to an Eigenvalue > 1 criterion, resulting in 23 extracted factors. Pearson’s correlations were then run between the factor scores of the first eight high-variance components deemed meaningful (consistent with a Scree test criterion and, respectively, accounting for at least 3% of the total variance after Varimax rotation) and the eight excluded items comprising Religious Coping and Religious Support (i.e., for the subset of participants who provided responses to these items). As these correlations are essentially equivalent to the factor loadings of the individual items if they had been part of the original PCA, this provided a convenient means to gauge the relationship of these additional variables to the R/S importance item. This combined data-driven approach allowed for a comprehensive evaluation of the interrelationship between the different religious scales and instruments with a special emphasis on their association with the R/S importance item.

To test the robustness of the PCA solution with regard to the High Risk design, analogous PCA solutions were obtained for critical subgroups, that is, for generations 2 (*n* = 140) and 3 (*n* = 99), and for high (*n* = 150) and low (*n* = 89) risk subgroups. Tucker’s factor congruence coefficient [43] was used to establish “equality” (≥ .95) or “fair similarity” (≥ .85) of factor loadings. To accomplish this, Tucker’s congruence coefficient was computed for pairwise comparisons of all factors of the original PCA solution (*N* = 282) with all factors of each of the four additional subgroup PCA solutions. Factors were deemed *robust* if they had a single corresponding factor matching *equality* in *all* subgroup PCA comparisons, or *almost robust* if unique factor correspondence reached at least *fair similarity*. For robust and almost robust factors, the corresponding factor scores were submitted to an analysis of covariance (i.e., one for each factor), using *generation* (2, 3) and *risk* (high, low) as between-subject variables, and gender and age as covariates. The analyses allowed evaluating whether stable latent factors stemming from the survey variables differed among offspring of the original probands as a function of familial risk status and generation.

Tucker’s factor congruence coefficient was also used to compare the PCA solutions obtained when using Varimax versus Promax rotations.

## Results

Table 2 summarizes the sample characteristics, including demographics, clinical and religious characteristics at the 35-year follow-up. Significant differences in frequency distributions of these variables between G2 and G3, which were the focus of this report, are reported in the last column of Table 2. The majority of the sample were female (60.6%), Catholic (49.1%), and from families at high risk for depression (63.1%). The oldest generation (G1) rated R/S Importance as being highly important more often than did the following generations (58.5% [G1] compared to 27.1% [G2] and 21.2% [G3]). Similarly, older generations attended weekly religious services more frequently than subsequent younger generations (46.3% [G1] as compared to 13.6% [G2] and 9.1% [G3]); this effect was also observed when directly comparing G2 and G3. Finally, the oldest generation (G1) identified primarily as being “religious and spiritual” (61%), whereas G2 and G3 were fairly evenly divided between identifying as “religious and spiritual” and “spiritual, but not religious.” As for a lifetime history of clinical diagnoses, G2 had significantly higher rates of depression and alcohol/drug disorders than G3, presumably because they were older and had more time to develop the disorders.

**Table 2:** Demographic, clinical, and religiosity characteristics of families at high and low risk for depression at most recent wave (Yr35).

Characteristics	Total *N* = 282*	Generation 1 *n* = 41	Generation 2 *n* = 141	Generation 3 *n* = 99	G2 vs. G3 ^ɸ^		
Age [Mean (SD)]	46.61 (17.91)	76.26 (0.49)	51.72 (7.48)	27.34 (5.88)	*χ*^2^	*df*	*p*
	N (%)	N (%)	N (%)	N (%)			
Gender							
Male	111 (39.4)	16 (39.0)	52 (36.9)	42 (42.4)	.75	1	.39
Female	171 (60.6)	25 (61.0)	89 (62.4)	57 (57.6)			
Risk for Depression (MDD)							
Low Risk	104 (36.9)	15 (36.6)	50 (35.5)	39 (39.4)	.39	1	.54
High Risk	178 (63.1)	26 (63.4)	91 (64.5)	60 (60.6)			
Clinical Diagnoses (Lifetime)**							
MDD	117 (41.5)	15 (36.6)	78 (55.7)	24 (24.2)	23.48	1	< .001
Anxiety Disorder	116 (41.1)	12 (29.3)	68 (48.6)	36 (36.4)	3.52	1	.06
Alcohol/Drug Disorder	106 (37.6)	13 (31.7)	68 (48.6)	25 (25.3)	13.27	1	< .001
Disruptive Disorder	42 (14.9)	1 (2.4)	27 (19.3)	14 (14.1)	1.08	1	.30
Suicide Attempts	4 (1.4)	1 (2.4)	3 (2.1)	0 (0)	2.15	1	.14
Religiosity/Spirituality (R/S)							
Denomination							
Catholic	138 (49.1)	25 (61.0)	68 (48.2)	45 (45.5)	1.54	2	.46
Protestant	48 (17.1)	8 (19.5)	26 (18.4)	14 (14.1)			
Other	95 (33.8)	8 (19.5)	47 (33.3)	40 (40.4)			
R/S Importance							
High	83 (29.4)	25 (58.5)	38 (27.1)	21 (21.2)	2.70	3	.44
Moderate	84 (29.8)	10 (24.4)	46 (32.9)	28 (28.3)			
Slight	80 (28.4)	7 (17.1)	39 (27.9)	34 (34.3)			
Not at All	34 (12.1)	0 (0)	17 (12.1)	16 (16.2)			
Religious Service Attendance							
Once a Week	47 (16.7)	19 (46.3)	19 (13.6)	9 (9.1)	9.55	4	.05
Once a Month	38 (13.5)	4 (9.8)	23 (16.6)	11 (11.1)			
1–2 Times a Year	76 (27.0)	9 (22.0)	34 (24.3)	33 (33.3)			
< Once a Year	58 (20.6)	5 (12.2)	37 (26.4)	16 (16.2)			
Never	62 (22.0)	4 (9.8)	27 (19.3)	30 (30.3)			
Religious Identity							
Religious and Spiritual	113 (40.1)	25 (61.0)	49 (34.8)	39 (39.4)	1.28	3	.74
Spiritual, Not Religious	95 (33.7)	9 (22.0)	53 (37.6)	33 (33.3)			
Religious, Not Spiritual	22 (7.8)	4 (9.8)	9 (6.4)	9 (9.1)			
Neither Spiritual or Religious	49 (17.4)	2 (4.9)	28 (19.9)	18 (18.2)			

*Includes one participant from Generation 4

**The clinical diagnoses are not mutually exclusive; multiple diagnoses are possible.

^ɸ^ Pearson’s Chi-Square test statistics are reported to compare the sampling distributions for Generations 2 and 3 only.

S1 Table (see Supplement) displays the Spearman rank order correlations of the R/S Importance item at the most recent wave (Yr35) with all survey items. The purpose of a non-parametric ranking was to provide an intuitive understanding which survey items and R/S scales associate most strongly with R/S Importance. Several scale constructs correlated highly with R/S Importance, including (a) belief salience, (b) religious engagement, (c) religious coping, and (d) self-transcendence. By and large, all religious/spiritual constructs retained the same order across generations and risk groups. Importantly, several items failed to correlate with R/S Importance, including items related to contemplative practice, eco-awareness, spirituality in nature, social support, love, and gratitude. Again, this lack of correlation was observed in the overall sample, and held its consistency across generations and risk groups.

As a more stringent test of association between R/S Importance and the other R/S constructs measured in the survey, we conducted a Varimax-rotated PCA to reveal the latent factor structure underlying all survey items, that is, to determine the R/S items that load on an independent, orthogonal dimension alongside the single-item of R/S Importance. The PCA resulted in a 23-factor solution (Eigenvalue > 1; 71.5% explained variance) with each of the first 8 extracted factors accounting for at least 3% of the total variance (see Table 3). The factor containing the single item of R/S importance (to which we will refer to as the R/S Importance Factor) explained 15.8% of the overall variance after rotation (factor 1) and had high internal consistency (Cronbach’s alpha = .84). Similar to the highest Spearman rank order correlations with R/S importance, the R/S Importance Factor included items related to Belief Salience, Religious Engagement, Religious Coping, Self-Transcendence, and Forgiveness by God. Apart from the R/S Importance Factor (factor 1), 7 other out of the 8 high-variance factors (> 3%) were readily interpretable and included, by order of extraction: (2) Spirituality in Nature, (3) Self-transcendence, (4) Altruism, (5) Love, (6) Gratitude, (7) Social Support, and (8) Mind Wandering.

**Table 3:** Varimax-rotated principal component loadings for questionnaire items at most recent wave (Yr35).

	Factor							
Items (*n* = 282 unless otherwise specified)	1	2	3	4	5	6	7	8
	R/S Importance	Spirituality in Nature	Self-transcendence	Altruism	Love	Gratitude	Social Support	Mind Wandering
BEL_SALIENCE_2	.**869**							
BEL_SALIENCE_3	.**852**							
BEL_SALIENCE_1	.**848**							
INTRINS_REL_2	.**825**							
R/S IMPORTANCE	.**819**							
INTRINS_REL_3	.**812**							
INTRINS_REL_1	.**799**							
REL_ENGAGE_1	-.**765**							
SELF_TRANS_16	.**745**							
BEL_SALIENCE_5	.**691**							
REL_ATTENDANCE	.**655**							
REL_ENGAGE_2	-.**654**							
REL_ENGAGE_3	-.**648**							
FORGIVE_3	.**646**							
SELF_TRANS_17	.**640**							
SELF_TRANS_4_Rev	.**583**							
SELF_TRANS_14	.**544**		.410					
SELF_TRANS_7	.**544**		.400					
SP_NATURE_3		.**820**						
SP_NATURE_4		.**811**						
ECO_AWARE_1		.**798**						
SP_NATURE_1		.**766**						
SP_NATURE_6		.**760**						
SP_NATURE_5		.**717**						
SP_NATURE_2	.334	.**670**						
SP_NATURE_7	.385	.**609**						
ECO_AWARE_6		.**564**	.464					
ECO_AWARE_2		.**551**						
SELF_TRANS_3		.**538**	.**530**					
SELF_TRANS_5			.**743**					
SELF_TRANS_8			.**686**					
SELF_TRANS_10			.**626**					
SELF_TRANS_18			.**609**					
SELF_TRANS_6			.**592**					
SELF_TRANS_2		.334	.**558**					
SELF_TRANS_25	.333		.**550**					
SELF_TRANS_13			.**522**					
SELF_TRANS_1			.**514**				.383	
SELF_TRANS_11			.396					
SELF_TRANS_15			.387					.351
ALTRUISM_1				.**785**				
ALTRUISM _2				.**765**				
ALTRUISM_4				.**756**				
ALTRUISM_3				.**664**				
ALTRUISM_6				.**608**				
ALTRUISM_5				.**517**			.381	
PSYC_LOVE_2					.**795**			
ONT_LOVE_1					.**736**			
ONT_LOVE_4					.**662**			
PSYC_LOVE_1					.**637**			
ONT_LOVE_2		.338	.334		.**612**			
PSYC_LOVE_4					.**602**			
ONT_LOVE_3					.438			
GRATITUDE_2						.**834**		
GRATITUDE_1						.**786**		
GRATITUDE_3						.**781**		
GRATITUDE_4						.**729**		
SOC_SUPPORT_3							.**835**	
SOC_SUPPORT_1							.**776**	
SOC_SUPPORT_2							.**769**	
PSYC_LOVE_3							.**519**	
SELF_TRANS_24								.**771**
SELF_TRANS_19								.**744**
SELF_TRANS_12								.**726**
SELF_TRANS_9			.367					.**600**
UNIVERSALITY_7	.473							
UNIVERSALITY_6	.478							
UNIVERSALITY_5	.363							
COMPASSION_2				.305				
ECO_AWARE_4		.353						
REL_ENGAGE_4	-.**594**							
SELF_TRANS_23_Rev	.396							
BEL_SALIENCE_4	.**548**							
SELF_TRANS_21			.339					.351
SELF_TRANS_20		.331						
REL_COPING_1* (*n* = 139)	.408							
REL_COPING_2* (*n* = 139)	.430							
REL_COPING_3* (*n* = 139)	.437							
REL_COPING_4* (*n* = 139)	.592							
REL_COPING_5* (*n* = 139)	.606							
REL_COPING_6* (*n* = 138)	.579							
REL_SUPPORT_1* (*n* = 126)	.318							
REL_SUPPORT_2* (*n* = 126)	.285							

Correlations in **bold** are loadings > .5. Loadings < .3 are not listed.

N.B. Religious Engagement (REL_ENGAGE) items were scored on a scale ranging from Highest to Lowest, rather than Lowest to Highest, as were the remaining scales.

* Variable/item not included in PCA due to missing data. Values listed are Pearson’s correlations, which are in this case equivalent to factor loadings.

Five of these 8 high-variance factors were also found to be consistent across the four identified subgroups: high vs. low risk, and generation 2 vs. 3 (see Supplemental S2 Table). The comparison of the factor loadings stemming from the subgroup PCA solutions via Tucker’s congruence coefficient (ϕ) indicated that factors 1 (R/S Importance Factor) and 2 (Spirituality in Nature Factor) were robust (*equality* correspondence of factor loadings in each subgroup), and factors 4 (Altruism Factor), 6 (Gratitude Factor) and 7 (Social Support Factor) were almost robust (at least *fair similarity* correspondence in each subgroup).

The ensuing repeated measures ANOVAs for the corresponding factor scores revealed significant effects only for the R/S Importance Factor: a generation main effect, *F*_[1,233]_ = 6.43, *p* = 0.01, stemming from greater R/S importance for generation 2 than 3, and a risk main effect, *F*_[1,233]_ = 7.18, *p* = 0.008, with low risk individuals having greater R/S importance than high risk individuals; however, there was no significant generation x risk interaction (*F*_[1,233]_ < 1.0). No significant main or interactions effects were observed for the other four factors deemed robust or almost robust (i.e., Spirituality in Nature Factor, Altruism Factor, Gratitude Factor, and Social Support Factor). This supports the notion that the R/S Importance Factor is a stable and reliable factor, one which allows for meaningful interpretability of results, and which differs between G2 and G3 and between low and high risk individuals.

Finally, the Promax-rotated PCA solution, which allowed for correlated factors, was remarkably similar to the Varimax-PCA solution (see Supplement S3 Table). All 23 extracted factors showed a 1:1 correspondence of at least *fair similarity* (0.88 ≤ ϕ ≤ 0.98) between solutions. Accordingly, the 8 high-variance factors identified by the Varimax rotation loaded highly and by-and-large on the same variables, thereby resulting in virtually the same factor structure.

## Discussion

To better understand the mechanisms implicated in the demonstrated protective effects on health when affirmatively answering the question how important to you is religion or spirituality [2–16], the present report assessed latent psychological components comprising a comprehensive survey of religious beliefs and experiences: R/S importance emerged as the primary factor. Several religious and spiritual constructs, as defined by existing instruments, correlated highly with the single-item measure of R/S importance across generation and familial risk status, namely, salient R/S beliefs that were internalized and personally experienced, as well as externalized religious practices (prayer, service attendance). Additionally, a personal relationship with the Divine played a central role in perceived R/S importance, such as having a personal relationship with God, feeling forgiven by God, and leaning on God through times of stress. These findings suggest that these particular characteristics of religiosity/spirituality are implicated most strongly in its protective effects, although future work will need to address this hypothesis directly.

Notably, not all previously defined sub-constructs of R/S correlated highly or consistently with R/S importance (i.e., both the single-item measure and the corresponding PCA component identified here). For example, measures of love, altruism, gratitude, contemplative/meditative practice, communing with nature, and social support were weakly correlated with R/S importance and, accordingly, loaded on separate PCA factors. This suggests that these aspects of religion, spirituality, and positive psychology, while important psychological constructs in and of themselves, may contribute minimally to the protective effects of R/S importance observed in previous studies.

Critically, the PCA solution obtained for the full sample was stable when compared to PCA solutions obtained for important subgroups of this cohort of families at risk for depression. Several factors were either robust (R/S Importance Factor, Spirituality in Nature Factor) or almost robust (Altruism Factor, Gratitude Factor, Social Support Factor), strongly suggesting that these represent genuine latent R/S constructs or constituents. However, only the R/S Importance Factor revealed differences between the cohort subgroups that are consistent with previously reported findings—that is, greater ratings of R/S importance in generation 2 than 3 [14] and in low than in high risk participants [11].

The factor structure observed here appeared to closely match the common dimensions of spirituality reported by McClintock et al. [28], which were religious commitment, contemplative practice, interconnectedness, love, and altruism. Their five-dimensional structure was uncovered in a large sample (*N* = 5512) of cross-cultural spirituality involving participants from China, India, and the United States across various religious backgrounds (Buddhism, Christianity, Hinduism, Islam, Judaism, non-religious, or other). Initially, the authors employed a data-driven approach on two subsamples (i.e., exploratory factor analyses with oblique rotation), which was then followed-up by confirmatory factor analyses on two other subsamples (i.e., validating the original factor structure via exploratory structural equation modeling), which suggested that these five dimensions of spirituality were universal (i.e., present across cultures, religions and societies). In a recent study relying virtually on the same participants included in the present report, McClintock et al. [27] were able to further support the validity of their five-factor structure when again relying on confirmatory factor analyses, comparing the present high and low risk sample (*N* = 281) to a Caucasian Christian American subsample (*N* = 602) from their prior study. The present study differed from McClintock et al. [27] in two critical aspects: 1) it included all of the data collected in the survey (i.e., it was not restricted to the subset of measures that had been included in the earlier McClintock et al. [28] study); and 2) it employed a fully data-driven approach with all survey items, as opposed to being confined by an a priori factor solution. While a detailed discussion of the nuanced differences between the multivariate data-reduction techniques employed in these two reports, which may have contributed to any differences in the factor structure, is beyond the scope of the present paper, it is nevertheless reassuring that several factors revealed a close correspondence, particularly the present R/S Importance Factor and McClintock et al.’s R/S Reflection and Commitment factor, with both factors sharing high loadings on the same survey items (e.g., intrinsic religiosity, belief salience, religious engagement). The present R/S Importance Factor, however, also included religious coping, certain aspects of self-transcendence, and forgiveness by God, all survey items that were not included by McClintock et al. [27–28]. Our other factors were seemingly consistent with most of the independent factors observed by McClintock et al. [28], with the exception of Contemplative Practice, the reasons of which are not immediately clear.

Interestingly, McClintock et al. [28] found that in the USA and India, individuals scoring in the top quartile of the R/S commitment factor (the one that correlated highly with R/S importance) were about 50% less likely to experience major depressive disorder, suicidal thoughts, and generalized anxiety disorder. Moreover, in our High Risk sample, McClintock et al. [27] found that previous diagnoses of major depression were associated with lower R/S commitment scores in high risk individuals, lower contemplation scores in low risk individuals, and lower R/S importance scores across both risk groups. These findings support our proposal that the protective effects of R/S importance are limited to only certain aspects of R/S (primarily, a personal relationship with the Divine and engagement with religious practices) and preclude others (i.e., those that load on other factors—gratitude, love, social support, altruism and spirituality in nature). The consistencies and discrepancies in factor structure between the present and the two McClintock et al. reports underscore the importance of the R/S variable selection as the most crucial decision when employing factor analytic techniques. By contrast, the extracted factor structure was not dependent on the choice of component rotation (i.e., orthogonal vs. oblique); rather, the present factor structure was evidently stable and primarily determined by the given set of input variables.

It is also worth noting that the single item of R/S Importance loaded on the same factor as Religious Service Attendance (another widely used single item measure used in mental health research). These two items were moderately correlated at *rho* = .52 (*p* < .01). Although it is true that people who reported religion or spirituality as being highly important to them were also more likely to attend religious services with greater frequency, this was not always the case and suggests that R/S importance encompasses something more than attendance at religious services. At the same time, religious attendance evidently contributes to what renders R/S as being personally important. Critically, only R/S importance was protective against depression in our previous studies, but not religious attendance [8–14]. The present findings hold important implications for health psychologists, psychiatrists, and clinicians in the interpretation and application of R/S importance in relation to health outcomes. The R/S importance item includes both internal (e.g., intrinsic religiosity, belief salience) and external aspects of religion and spirituality (engaging in religious activities)–and not only internal components as is often suggested [2]. Importantly, this single item appears to relate specifically to the heart of the individual through a personal belief in and relationship with God, one that is externalized through religious practices. On the other hand, the single item measure evidently does not capture other aspects of spirituality (e.g., communion with nature, gratitude, altruism). Of note, the possibility that religiosity exceeds spirituality in its protective effects against depression was also observed in several longitudinal studies [44–46].

Limitations of the present study include the cross-sectional design of the extensive array of R/S measures with clinical diagnoses. It should also be noted that the sample upon which the study is based was predominantly Caucasian and Catholic; nonetheless, given the universality of the common R/S dimensions reported by McClintock et al. [28] and replicated in this same sample [27], this may not be a crucial limitation. Still, further research is needed to determine whether our results generalize to other faith traditions and religious groups. As discussed above, our findings are also naturally limited by the constructs and subscales employed in our survey despite the effort to employ a comprehensive selection of R/S scales.

We also note the limitation of the single-item measure of R/S importance itself. More complex measures may distinguish between religiosity and spirituality, whereas religiosity involves formal or informal religious practices (public or private), spirituality involves the individual's relationship to a transcendent force (God or higher power [47]; cf. [48]). Spirituality is said to represent an integrative force in the individual's life [49], providing meaning, core values, and principles for organizing one's life, which may or may not be a part of religion. To the extent that spirituality and religion constitute unique constructs, they are necessarily conflated by a single-item measure.

As another possible limitation, the sample size of *N* = 282 may be too small for a PCA with 101 variables because it violates a popular rule calling for a cases-to-variables ratio of no less than 5:1, although this rule—without further qualification—lacks both empirical support and a theoretical rationale [50–51]. For the current data, 7 out of the 8 factors described in Table 3 included four or more variables with loadings above .6 (the R/S importance factor had 15), thereby warranting their interpretation independent of sample size [50]. More importantly, the systematic comparisons of PCA solutions using Tucker’s congruence coefficient identified several factors as being robust, including the R/S importance factor, despite the fact that each of the subgroup PCAs violated the item ratio rule to an even greater degree.

## Conclusion

Although using a multi-dimensional questionnaire to measure religiosity/spirituality may be desirable, time and energy-expenditure constrains feasibility of comprehensive instruments for various populations, community samples, and mental disorders alike. Our findings, however, strongly suggest that the single R/S importance item has adequate construct validity and may therefore be sufficient for many applied and basic research purposes. Our findings accordingly corroborate the item’s obvious face validity: it captures much of what may be intuitively conceived as personal R/S importance when individuals are asked—to provide an integrative summary estimate of the R/S construct—which is the intent in the first place. Accordingly, it also captures both internal and external aspects of religion and spirituality and maintains considerable consistency across generations and risk groups for familial depression. Nonetheless, it is important for researchers to keep in mind the specific aspects of R/S importance included and precluded by the single item, as detailed by this report, and to apply these considerations when interpreting future findings. As such, the single-item measure alone fails to capture important aspects of religiosity and spirituality, including spirituality experienced through nature, meditative practices, gratitude, love, and compassion. Moreover, theoretical distinctions between religiosity and spirituality remain undifferentiated by this single item, and whether specific activities and forms of religious or spiritual engagement provide protection against depression (and other mental health disorders) is left to future research using a broader conceptualization and measurement of the R/S construct.

## Supporting information

S1 Table Nonparametric Spearman rank-order correlations between religious/spiritual importance (at Yr35) and each quantitative variable included in the religiosity/spirituality (R/S) survey for full sample and subgroup categories reflecting risk status and generation. (PDF)

S2 Table Comparison of PCA solutions (factor loadings) using Tucker’s congruence coefficient. (PDF)

S3 Table Promax-rotated principal component loadings for questionnaire items at most recent wave (Yr35). (PDF)
