# Supplementary material for: Understanding self-reported importance of religion/spirituality in a North American sample of individuals at risk for familial depression: A principal component analysis
Source: PLoS One. 2019 Oct 18;14(10):e0224141. doi: 10.1371/journal.pone.0224141 (PMC6799910; doi:10.1371/journal.pone.0224141)
Supplement: S1 Table — (PDF) [file pone.0224141.s001.pdf]

S1 Table. Nonparametric Spearman rank-order correlations between religious/spiritual importance (at Yr35) and each quantitative variable included in the religiosity/spirituality (R/S) survey for full sample and subgroup categories reflecting risk status and generation.

All Quantitative Yr35 (Templeton Study) Variables							Correlation[b] of Each Quantitative Yr35 Variable With 'Current Religious/Spiritual Importance'									
							Yr35		High Risk		Low Risk		G2		G3	
Variable Name[a]	Label	*N*	*M*	SD	Min	Max	*rho*[c]	n	*rho*	n	*rho*	n	*rho*	n	*rho*	n
R/S_IMPORTANCE	Personal importance of religion/spirituality.	281	3.2	1	2	5	–	–	1	176	1	104	1	140	1	99
BEL_SALIENCE_3	I am frequently aware of God in a personal way.	281	4.8	2	1	7	-.75****	280	-.74****	175	-.78****	104	-.71****	139	-.76****	99
BEL_SALIENCE_1	My religious beliefs are what lie behind my whole approach to life.	281	4.2	1.9	1	7	-.74****	280	-.77****	175	-.67****	104	-.68****	139	-.81****	99
BEL_SALIENCE_2	My religious beliefs provide meaning and purpose to life.	281	4.5	1.9	1	7	-.73****	280	-.75****	175	-.68****	104	-.65****	139	-.80****	99
REL_ENGAGE_1	I pray privately (for myself, for others).	280	2.4	1.1	1	4	.71****	279	.73****	175	.64****	103	.71****	139	.67****	99
INTRINS_REL_2	My religious beliefs are what really lie behind my whole approach to life.	282	2.9	1.4	1	5	-.68****	281	-.72****	176	-.60****	104	-.69****	140	-.68****	99
INTRINS_REL_1	In my life, I experience the presence of the Divine (i.e., God).	278	3.2	1.3	1	5	-.67****	277	-.69****	173	-.62****	103	-.65****	138	-.69****	97
INTRINS_REL_3	I try hard to carry my religion over into all other dealings in life.	282	2.7	1.3	1	5	-.66****	281	-.64****	176	-.70****	104	-.63****	140	-.71****	99
REL_ENGAGE_3	In my private area, religious symbols are important to me.	281	3	1	1	4	.61****	280	-.58****	175	.66****	104	.56****	140	.65****	99
REL_COPING_5	Trusted that God would be by my side.	139	3.1	0.9	1	4	-.60****	138	-.62****	86	-.57****	52	-.61****	71	-.68****	40
SELF_TRANS _16	Religious experiences have helped me to understand the real purpose of my life.	281	2.7	1.4	1	5	-.60****	280	-.63****	175	-.53****	104	-.51****	140	-.71****	98
SELF_TRANS _17	I believe that all life depends on some spiritual order or power that cannot be completely explained.	282	3.2	1.4	1	5	-.58****	281	-.66****	176	-.40****	104	-.57****	140	-.54****	99
REL_ENGAGE_2	I go to church/mosque/synagogue (a religious house of worship).	281	2.9	1	1	4	.55****	280	-.53****	175	.59****	104	.50****	139	.57****	99
REL_COPING_6	Looked to God for strength, support, and guidance.	138	3.2	0.9	1	4	-.54****	137	-.53***	86	-.55****	51	-.56****	70	-.45***	40

****p < .0001. ***p < .005. **p < .01. *p < .05. *ns* = not significant.
[a]Only ordinal (including dichotomous) variables were eligible for this list. [b]Spearman rank-order correlation coefficient (*rho*). [c]The entire table is sorted for column Yr35 in descending order of absolute magnitude (i.e., "sign-free").

All Quantitative Yr35 (Templeton Study) Variables

Correlation[b] of Each Quantitative Yr35 Variable With 'Current Religious/Spiritual Importance'

							Yr35		High Risk		Low Risk		G2		G3	
**Variable Name[a]**	**Label**	***N***	***M***	**SD**	**Min**	**Max**	***rho*[c]**	**n**	***rho***	**n**	***rho***	**n**	***rho***	**n**	***rho***	**n**
REL_ENGAGE_4	I participate in religious events (e.g., religious congregatio*ns*).	277	3.1	0.9	1	4	.53****	276	.49****	173	.59****	102	.43****	138	.58****	98
REL_ATTENDANCE	Religious attendance (current -- reported by self at Wave 7)	281	3.8	1.4	2	6	-.52****	280	-.74****	175	-.60****	104	-.44****	139	-.51****	99
BEL_SALIENCE_5	Being a religious person is important to me.	279	4.2	1.9	1	7	-.52****	278	-.42****	173	-.68****	104	-.49****	138	-.53****	98
FORGIVE_3	I know that God forgives me.	275	3.1	0.9	1	4	-.50****	274	-.60****	172	-.31***	102	-.48****	135	-.52****	97
SELF_TRANS _4	I think that most things that are called miracles are just chance.	280	2.8	1.3	1	5	.50****	279	.62****	174	.27**	104	.53****	138	.47****	99
SELF_TRANS _7	Sometimes I have felt my life was being directed by a spiritual force greater than any human being.	281	2.9	1.4	1	5	-.50****	280	-.53****	176	-.43****	103	-.54****	139	-.52****	99
SELF_TRANS _14	I have had personal experiences in which I have felt in contact with a divine and wonderful spiritual power.	282	2.6	1.5	1	5	-.49****	281	-.58****	176	-.31***	104	-.51****	140	-.41****	99
REL_COPING_4	Sought God's love and care.	139	3	0.9	1	4	-.49****	138	-.54****	86	-.39***	52	-.42***	71	-.63****	40
UNIVERSALITY_6	I believe that death is a doorway to another plane of existence.	280	3.5	1.1	1	5	-.43****	279	-.45****	175	-.40****	103	-.40****	139	-.50****	98
UNIVERSALITY_7	I believe there is a larger plan to life.	280	3.6	1.1	1	5	-.41****	279	-.46****	174	-.30***	104	-.44****	138	-.40****	99
BEL_SALIENCE_4	I allow my religious beliefs to influence other areas of my life.	281	4	1.8	1	7	-.41****	280	-.36****	175	-.51****	104	-.41****	139	-.44****	99
REL_COPING_3	Tried to see how God might be trying to strengthen me in this situation.	139	2.8	1	1	4	-.38****	138	-.35***	86	-.44***	52	-.47****	71	-.51***	40
CONGREG_w7	Do you belong to a religious congregation (e.g., a church, synagogue)? (Wave 7)	282	1.5	0.5	1	2	.38****	281	.31****	1761	.51****	104	.32****	140	.39****	99
REL_COPING_2	Tried to find a lesson from God in the event.	139	2.6	1	1	4	-.38****	138	-.43****	86	-.29*	52	-.44****	71	-.47***	40
UNIVERSALITY_5	I believe that there is a larger meaning to life.	281	3.8	1	1	5	-.38****	280	-.45****	175	-.24*	104	-.44****	139	-.37***	99
SP_NATURE_7	Being in nature is my favorite place to talk to God.	281	2.7	1.1	1	5	-.37****	280	-.44****	176	-.25**	103	-.29***	139	-.35***	99

****p < .0001. ***p < .005. **p < .01. *p < .05. *ns* = not significant.
[a]Only ordinal (including dichotomous) variables were eligible for this list. [b]Spearman rank-order correlation coefficient (*rho*). [c]The entire table is sorted for column Yr35 in descending order of absolute magnitude (i.e., "sign-free").

							Correlation[b] of Each Quantitative Yr35 Variable With 'Current Religious/Spiritual Importance'									
All Quantitative Yr35 (Templeton Study) Variables							Yr35		High Risk		Low Risk		G2		G3	
**Variable Name[a]**	**Label**	***N***	***M***	**SD**	**Min**	**Max**	***rho*[c]**	**n**	***rho***	**n**	***rho***	**n**	***rho***	**n**	***rho***	**n**
SELF_TRANS _23	I think it is unwise to believe in things that cannot be explained scientifically.	281	2.5	1.3	1	5	.37****	280	.40****	176	.31***	103	.28***	140	.43****	98
SELF_TRANS_6	I sometimes feel a spiritual connection to other people that I cannot explain in words.	282	3.1	1.4	1	5	-.37****	281	-.40****	176	-.32***	104	-.47****	140	-.35***	99
REL_COPING_1	Saw my situation as part of God's plan.	139	2.5	1.1	1	4	-.35****	138	-.39***	86	-.26 *ns*	52	-.33**	71	-.64****	40
REL_SUPPORT_1	If you were ill, how much would the people in your congregation help you out?	126	2.6	1.1	1	4	-.35****	126	-.36***	75	-.37**	51	-.33**	62	-.52***	38
SP_NATURE_2	I se*n*se God's presence more in nature than I do anywhere else.	282	3.1	1.3	1	5	-.34****	281	-.38****	176	-.25*	104	-.31***	140	-.39****	99
REL_SUPPORT_2	If you had a problem or were faced with a difficult situation, how much comfort would the people in your congregation be willing to give you?	126	2.7	1	1	4	-.31***	126	-.34***	75	-.33*	51	-.32*	62	-.37*	38
SELF_TRANS _25	I have had experiences that made my role in life so clear to me that I felt very excited and happy.	281	2.9	1.3	1	5	-.31****	280	-.31***	176	-.31***	103	-.34****	140	-.37***	98
SELF_TRANS _8	I have had moments of great joy in which I suddenly had a clear, deep feeling of oneness with all that exists.	282	3	1.3	1	5	-.30****	281	-.29****	176	-.34***	104	-.44****	140	-.26**	99
SELF_TRANS _10	I often feel a strong spiritual or emotional connection with all the people around me.	281	2.8	1.2	1	5	-.29****	280	-.35****	176	-.19 *ns*	103	-.28***	139	-.35***	99
ALTRUISM_3	My thoughts are with those in need.	279	3.2	0.7	1	4	-.29****	278	-.27***	174	-.34***	103	-.30***	139	-.32***	97
SELF_TRANS _26	Reports of mystical experiences are just wishful thinking.	282	2.6	1.2	1	5	.29****	281	.32***	176	.24*	104	.26***	140	.29***	99
SELF_TRANS _15	Often when I look at an ordinary thing, something wonderful happe*ns* -- I get the feeling that I am seeing it fresh for the first time.	281	2.5	1.3	1	5	-.27****	280	-.24***	176	-.35***	103	-.25***	139	-.26**	99
SELF_TRANS _13	I often feel like I am a part of the spiritual force on which all life depends.	282	2.1	1.2	1	5	-.26****	281	-.38****	176	-.07 *ns*	104	-.35****	140	-.23*	99
UNIVERSALITY_9	I believe that on some level my life is intimately tied to all of humankind.	282	3.2	1	1	5	-.26****	281	-.29***	176	-.22*	104	-.15 *ns*	140	-.34***	99

****p < .0001. ***p < .005. **p < .01. *p < .05. *ns* = not significant.
[a]Only ordinal (including dichotomous) variables were eligible for this list. [b]Spearman rank-order correlation coefficient (*rho*). [c]The entire table is sorted for column Yr35 in descending order of absolute magnitude (i.e., "sign-free").

All Quantitative Yr35 (Templeton Study) Variables

Correlation[b] of Each Quantitative Yr35 Variable With 'Current Religious/Spiritual Importance'

							Yr35		High Risk		Low Risk		G2		G3	
**Variable Name[a]**	**Label**	***N***	***M***	**SD**	**Min**	**Max**	***rho*[c]**	**n**	***rho***	**n**	***rho***	**n**	***rho***	**n**	***rho***	**n**
COMPASSION_4	I feel sorry for someone who is in trouble even when they caused the problem that faces them.	280	2.7	0.7	1	4	-.25****	279	-.23***	175	-.30***	103	-.19*	139	-.30***	99
SELF_TRANS _18	I often feel so connected to the people around me that it is like there is no separation between us.	281	2.4	1.2	1	5	-.24****	280	-.33****	175	-.06 *ns*	104	-.31***	140	-.17 *ns*	98
CONT_PRACT_Medit_Days_w6b	On average, how often do you meditate, in terms of days per month?	273	2.9	7.2	0	30	-.24****	272	-.28***	172	-.17 *ns*	99	-.24**	134	-.16 *ns*	98
VOLUNTEER	How often do you spend working time in programs, whether through a religious organization or not.	281	5	1.4	1	6	.23****	280	.20**	175	.31***	104	.22*	140	.24*	99
UNIVERSALITY_3	There is a higher plane of consciousness or spirituality that binds all people.	280	3.3	1	1	5	-.22***	279	-.30****	174	-.06 *ns*	104	-.24***	139	-.22*	98
COMPASSION_3	It's not enough to feel sorry for someone who is in trouble: Whenever it is possible, I must also do something to help them.	281	2.9	0.7	1	4	-.22***	280	-.28***	175	-.13 *ns*	104	-.18*	139	-.29***	99
ONT_LOVE_4	Love for love's sake brings the greatest happiness.	282	3.8	0.9	1	5	-.21***	281	-.29****	176	-.11 *ns*	104	-.11 *ns*	140	-.37***	99
ALTRUISM_5	I feel connected with others.	281	3.1	0.7	1	4	-.21***	280	-.27***	175	-.08 *ns*	104	-.12 *ns*	140	-.27**	98
SELF_TRANS _1	I often feel a strong sense of unity with all the things around me.	280	3.6	1	1	5	-.20***	279	-.26***	175	-.07 *ns*	103	-.19*	139	-.19 *ns*	99
UNIVERSALITY_8	There is an order to the universe that transcends human thinking.	282	3.6	0.9	1	5	-.20***	281	-.22***	176	-.20*	104	-.09 *ns*	140	-.27**	99
FORGIVE_2	I have forgiven those who have hurt me.	281	2.9	0.6	1	4	-.20***	280	-.18*	175	-.25*	104	-.09 *ns*	139	-.29***	99
SOC_LOVE_4	The best kind of love is given freely.	279	4.3	0.7	1	5	-.20**	278	-.23***	174	-.15 *ns*	103	-.17 *ns*	137	-.20*	99
CONT_PRACT_Medit_Mos_w6b	How many months have you been practicing meditation?	263	9.9	36.2	0	324	-.19**	262	-.24***	163	-11 *ns*	98	-.24**	131	-.16 *ns*	98
ALTRUISM_1	I help others.	282	3.5	0.6	2	4	-.19**	281	-.22***	176	-.14 *ns*	104	-.16 *ns*	140	-.23*	99
PSYC_LOVE_1	Feeling loved is my greatest source of happiness.	281	4	0.9	1	5	-.19**	280	-.30****	175	.00 *ns*	104	-.14 *ns*	140	-.30***	98

****p < .0001. ***p < .005. **p < .01. *p < .05. *ns* = not significant.
[a]Only ordinal (including dichotomous) variables were eligible for this list. [b]Spearman rank-order correlation coefficient (*rho*). [c]The entire table is sorted for column Yr35 in descending order of absolute magnitude (i.e., "sign-free").

All Quantitative Yr35 (Templeton Study) Variables

Correlation[b] of Each Quantitative Yr35 Variable With 'Current Religious/Spiritual Importance'

							Yr35		High Risk		Low Risk		G2		G3	
**Variable Name[a]**	**Label**	***N***	***M***	**SD**	**Min**	**Max**	***rho*[c]**	**n**	***rho***	**n**	***rho***	**n**	***rho***	**n**	***rho***	**n**
SOC_LOVE_2	Even strangers deserve our full respect.	282	4	0.8	1	5	-.18**	281	-.23***	176	-.10 *ns*	104	-.14 *ns*	140	-.35***	99
ECO_AWARE_2	I believe there is a connection between all living things that I cannot see but can sense.	280	4.1	1.4	1	6	-.18**	279	-.25***	174	-.07 *ns*	104	-.25***	139	-.07 *ns*	99
ONT_LOVE_2	Experiencing love helps me feel at one with the universe.	282	3.4	1	1	5	-.18**	281	-.22***	176	-.10 *ns*	104	-.12 *ns*	140	-.23*	99
COMPASSION_1	When I see someone in a difficult situation I try to imagine how they feel.	281	3.3	0.6	1	4	-.18**	280	-.20**	175	-.16 *ns*	104	-.27***	139	-.09 *ns*	99
SELF_TRANS _2	Often I have unexpected flashes of insight or understanding while relaxing.	282	3.4	1.2	1	5	-.18**	281	-.15*	176	-.25**	104	-.27***	140	-.06 *ns*	99
ONT_LOVE_1	When I feel loved, I feel complete peace of mind.	282	3.9	0.9	1	5	-.17**	281	-.18*	176	-.13 *ns*	104	-.06 *ns*	140	-.32***	99
ALTRUISM_2	I consider the needs of others.	281	3.5	0.6	2	4	-.17**	280	-.19*	176	-.13 *ns*	103	-.22*	140	-.11 *ns*	99
GRATITUDE_2	If I had to list everything that I felt grateful for, it would be a very long list.	281	3.5	0.7	1	4	-.17**	280	-.23**	176	-.05 *ns*	103	-.16 *ns*	140	-.13 *ns*	99
UNIVERSALITY_4	Although individual people may be difficult, I feel an emotional bond with all of humanity.	281	3.1	1	1	5	-.16**	280	-.17*	175	-.15 *ns*	104	-.12 *ns*	140	-.16 *ns*	99
SELF_TRANS _3	I sometimes feel so connected to nature that everything seems to be part of one living process.	282	3.2	1.2	1	5	-.16**	281	-.14 *ns*	176	-.20*	104	-.24***	140	-.05 *ns*	99
ALTRUISM_6	I work voluntarily for others.	282	2.9	0.9	1	4	-.15*	281	-.21**	176	-.01 *ns*	104	-.11 *ns*	140	-.13 *ns*	99
ONT_LOVE_3	When I'm kind, good things usually happen to me in return.	282	3.7	1	1	5	-.14*	281	-.17*	176	-.10 *ns*	104	-.08 *ns*	140	-.19 *ns*	99
GRATITUDE_1	I have much in life to be grateful for.	282	3.7	0.5	1	4	-.13*	281	-.16*	176	-.06 *ns*	104	-.15 *ns*	140	-.12 *ns*	99
SP_NATURE_6	Experiences in nature have fostered my spiritual growth.	281	2.9	1.2	1	5	-.13*	280	-.19*	175	-.05 *ns*	104	-.12 *ns*	140	-.07 *ns*	98
SOC_LOVE_1	I have always been a devoted friend.	282	4.1	0.9	1	5	-.13*	281	-.17*	176	-.02 *ns*	104	-.06 *ns*	140	-.27**	99
SOC_LOVE_3	For a friend in need, I would sacrifice almost anything.	282	3.8	0.9	1	5	-.12*	281	-.15 *ns*	176	-.07 *ns*	104	-.23**	140	-.12 *ns*	99

****p < .0001. ***p < .005. **p < .01. *p < .05. *ns* = not significant.
[a]Only ordinal (including dichotomous) variables were eligible for this list. [b]Spearman rank-order correlation coefficient (*rho*). [c]The entire table is sorted for column Yr35 in descending order of absolute magnitude (i.e., "sign-free").

All Quantitative Yr35 (Templeton Study) Variables							Correlation[b] of Each Quantitative Yr35 Variable With 'Current Religious/Spiritual Importance'									
							Yr35		High Risk		Low Risk		G2		G3	
**Variable Name[a]**	**Label**	***N***	***M***	**SD**	**Min**	**Max**	***rho*[c]**	**n**	***rho***	**n**	***rho***	**n**	***rho***	**n**	***rho***	**n**
SP_NATURE_3	I usually feel more comfortable talking/thinking through my problems in nature.	282	3.2	1.1	1	5	-.12*	281	-.09 *ns*	176	-.21*	104	-.12 *ns*	140	-.03 *ns*	99
PSYC_LOVE_2	Feeling loved takes away all my fear.	282	3.5	1	1	5	-.12 *ns*	281	-.19*	176	.03 *ns*	104	-.10 *ns*	140	-.13 *ns*	99
SELF_TRANS _22	I would gladly risk my own life to make the world a better place.	280	2.8	1.2	1	5	-.11 *ns*	279	-.17 *	175	-.02 *ns*	103	-.14 *ns*	138	-.05 *ns*	99
PSYC_LOVE_4	Without having others to love, life wouldn't be worth living.	281	3.6	1.1	1	5	-.11 *ns*	280	-.15 *ns*	175	-.06 *ns*	104	-.07 *ns*	139	-.18 *ns*	99
SP_NATURE_5	I feel more appreciated in nature than I do anywhere else.	282	2.7	1.1	1	5	-.10 *ns*	281	-.10 *ns*	176	-.12 *ns*	104	-.05 *ns*	140	-.13 *ns*	99
SOC_SUPPORT_2	How often do your family and friends talk with you about your private problems and concer*ns*?	282	2.9	0.9	1	4	-.10 *ns*	281	-.14 *ns*	176	-.02 *ns*	104	-.08 *ns*	140	-.13 *ns*	99
ALTRUISM_4	I do good.	281	3.5	0.5	2	4	-.09 *ns*	280	-.14 *ns*	175	-.01 *ns*	104	.02 *ns*	140	-.24*	99
SP_NATURE_4	Spending time in nature helps me to understand my purpose in life.	281	3	1.1	1	5	-.08 *ns*	280	-.02 *ns*	176	-.21*	103	-.05 *ns*	140	-.05 *ns*	98
ECO_AWARE_5	I believe that nature should be respected.	280	5.4	0.7	4	6	.08 *ns*	279	.08 *ns*	175	.05 *ns*	103	.14 *ns*	140	.05 *ns*	98
ECO_AWARE_6	At times, I feel at one with the universe.	280	3.5	1.4	1	6	-.08 *ns*	279	-.09 *ns*	175	-.07 *ns*	103	-.06 *ns*	140	-.12 *ns*	99
GRATITUDE_3	I am grateful to a wide variety of people.	282	3.4	0.7	1	4	-.08 *ns*	281	-.15*	176	.06 *ns*	104	-.04 *ns*	140	-.18 *ns*	99
COMPASSION_2	I feel compelled to help someone even when doing so requires me to go out of my way.	281	3.2	0.7	1	4	-.07 *ns*	280	-.08 *ns*	175	-.04 *ns*	104	-.07 *ns*	139	-.07 *ns*	99
UNIVERSALITY_1	I feel that on a higher level all of us share a common bond.	281	3.5	1	1	5	-.07 *ns*	280	-.08 *ns*	175	-.07 *ns*	104	-.04 *ns*	140	-.04 *ns*	99
SOC_SUPPORT_1	How often do your family members and friends let you know they love and care for you?	282	3.4	0.8	1	4	-.07 *ns*	281	-.16*	176	.09 *ns*	104	-.11 *ns*	140	-.02 *ns*	99
SELF_TRANS _20	I often do things to help protect animals and plants from extinction.	282	2.9	1.3	1	5	-.06 *ns*	281	-.04 *ns*	176	-.11 *ns*	104	.02 *ns*	140	-.06 *ns*	99
SELF_TRANS _19	I am often called 'absent-minded' because I get so wrapped up in what I am doing that I lose track of everything else.	282	2	1.2	1	5	-.06 *ns*	281	-.02 *ns*	176	-.12 *ns*	104	-.06 *ns*	140	-.12 *ns*	99

****p < .0001. ***p < .005. **p < .01. *p < .05. *ns* = not significant.

[a]Only ordinal (including dichotomous) variables were eligible for this list. [b]Spearman rank-order correlation coefficient (*rho*). [c]The entire table is sorted for column Yr35 in descending order of absolute magnitude (i.e., "sign-free").

All Quantitative Yr35 (Templeton Study) Variables

Correlation[b] of Each Quantitative Yr35 Variable With ‘Current Religious/Spiritual Importance’

							Yr35		High Risk		Low Risk		G2		G3	
**Variable Name[a]**	**Label**	***N***	***M***	**SD**	**Min**	**Max**	***rho*[c]**	**n**	***rho***	**n**	***rho***	**n**	***rho***	**n**	***rho***	**n**
SELF_TRANS _11	I have made real personal sacrifices in order to make the world a better place --like trying to prevent war, poverty, and injustice.	282	2.4	1.2	1	5	-.05 *ns*	281	-.10 *ns*	176	.03 *ns*	104	-.06 *ns*	140	.05 *ns*	99
PSYC_LOVE_3	As long as I can remember, I have always been loved.	280	4	1	1	5	.05 *ns*	279	-.04 *ns*	176	.24*	102	.04 *ns*	139	.03 *ns*	99
SELF_TRANS _5	Sometimes I have felt like I was part of something with no limits or boundaries in time and space.	280	2.7	1.2	1	5	-.05 *ns*	279	-.02 *ns*	176	-.13 *ns*	102	-.11 *ns*	139	.01 *ns*	99
SELF_TRANS _12	It often seems to other people like I am in another world because I am so completely unaware of things going on around me.	282	1.8	1.2	1	5	.04 *ns*	281	.10 *ns*	176	-.10 *ns*	104	-.02 *ns*	140	.04 *ns*	99
ECO_AWARE_4	The earth is sacred.	281	5	1.1	1	6	-.04 *ns*	280	-.07 *ns*	175	.01 *ns*	104	.07 *ns*	139	-.18 *ns*	99
FORGIVE_1	I have forgiven myself for things I have done wrong.	281	3	0.7	1	4	-.04 *ns*	280	-.06 *ns*	175	.01 *ns*	104	.06 *ns*	139	-.14 *ns*	99
UNIVERSALITY_2	All life is interconnected.	282	3.7	0.9	1	5	-.04 *ns*	281	-.07 *ns*	176	.02 *ns*	104	-.03 *ns*	140	.00 *ns*	99
ECO_AWARE_1	I feel in harmony with nature.	276	4	1.3	1	6	-.03 *ns*	275	-.04 *ns*	174	-.03 *ns*	100	-.02 *ns*	138	.03 *ns*	98
CONT_PRACT_MindBody _Days_w6b	On average, how often do you do a mind-body practice (yoga, qigong, etc.), in terms of days per month?	272	1.7	4.9	0	30	.03 *ns*	271	.01 *ns*	168	.05 *ns*	102	-.02 *ns*	134	-.02 *ns*	98
SOC_SUPPORT_3	How often do your family and friends express interest and concern in your well-being?	280	3.1	0.9	1	4	-.03 *ns*	279	-.12 *ns*	175	.13 *ns*	103	-.01 *ns*	140	-.02 *ns*	99
SELF_TRANS _24	Often I become so involved in what I am doing that I forget where I am for a while.	280	2.1	1.2	1	5	.01 *ns*	279	.01 *ns*	174	.01 *ns*	104	-.03 *ns*	138	-.05 *ns*	99
CONT_PRACT_MindBody _Mos_w6b	How many months have you been doing mind-body practices?	267	9.5	34.1	0	300	.01 *ns*	266	-.06 *ns*	166	.13 *ns*	99	-.05 *ns*	132	-.05 *ns*	98
ECO_AWARE_3	I believe that all living creatures deserve respect.	281	5.1	1	1	6	-.01 *ns*	280	-.03 *ns*	176	.01 *ns*	103	.03 *ns*	140	.00 *ns*	99
SELF_TRANS _21	I have a vivid imagination.	281	3.4	1.2	1	5	.00 *ns*	280	-.02 *ns*	176	.03 *ns*	104	.05 *ns*	139	-.11 *ns*	99
SELF_TRANS _9	I often become so fascinated with what I'm doing that I get lost in the moment -- like I'm detached from time and place.	282	2.9	1.3	1	5	.00 *ns*	281	.09 *ns*	176	-.17 *ns*	104	-.11 *ns*	140	-.02 *ns*	99

****p < .0001. ***p < .005. **p < .01. *p < .05. *ns* = not significant.
[a]Only ordinal (including dichotomous) variables were eligible for this list. [b]Spearman rank-order correlation coefficient (*rho*). [c]The entire table is sorted for column Yr35 in descending order of absolute magnitude (i.e., “sign-free”).

****p < .0001. ***p < .005. **p < .01. *p < .05. *ns* = not significant.
[a]Only ordinal (including dichotomous) variables were eligible for this list. [b]Spearman rank-order correlation coefficient (*rho*). [c]The entire table is sorted for column Yr35 in descending order of absolute magnitude (i.e., “sign-free”).
