# Supplementary material for: Understanding self-reported importance of religion/spirituality in a North American sample of individuals at risk for familial depression: A principal component analysis
Source: PLoS One. 2019 Oct 18;14(10):e0224141. doi: 10.1371/journal.pone.0224141 (PMC6799910; doi:10.1371/journal.pone.0224141)
Supplement: S2 Table — (PDF) [file pone.0224141.s002.pdf]

S2 Table. Comparison of PCA solutions (factor loadings) using Tucker's Congruence Coefficient.

Generation 2 subset (*n* = 141)

	1	2	3	4	5	6	7	8	9	10
1	**0.988**	0.237	0.344	0.286	0.239	0.212	0.267	0.209	0.370	0.081
2	0.237	**0.978**	0.524	0.352	0.192	0.112	0.114	0.206	0.366	0.187
3	0.378	0.603	**0.943**	0.440	0.285	0.269	0.216	0.187	0.465	0.283
4	0.248	0.195	0.252	0.293	**0.968**	0.224	0.188	0.409	0.279	0.040
5	0.258	0.360	0.286	**0.941**	0.289	0.367	0.231	0.263	0.258	0.100
6	0.249	0.099	0.174	0.242	0.175	0.519	**0.956**	0.209	0.193	-0.110
7	0.170	0.097	0.218	0.363	0.224	**0.926**	0.495	0.097	0.180	-0.057
8	0.117	0.264	0.362	0.129	0.047	-0.036	-0.105	0.154	0.140	**0.923**
9	0.391	0.330	0.364	0.130	0.194	0.195	0.184	0.228	0.805	0.080
10	0.224	0.407	0.369	0.194	0.205	0.211	0.171	0.091	0.695	0.027

Generation 3 subset (*n* = 99)

	1	2	3	4	5	6	7	8	9	10
1	**0.978**	0.181	0.439	0.204	0.138	0.287	0.204	0.176	0.287	0.034
2	0.181	**0.960**	0.480	0.429	-0.006	0.447	0.159	0.016	0.273	0.309
3	0.271	0.626	0.422	0.846	0.071	0.721	0.214	0.098	0.327	0.393
4	0.276	0.236	0.236	0.189	0.052	0.179	**0.908**	0.168	0.208	0.169
5	0.309	0.290	0.255	0.360	0.185	0.259	0.269	0.247	**0.923**	0.147
6	0.288	-0.004	0.127	0.166	0.484	0.118	0.146	**0.882**	0.213	-0.090
7	0.191	0.035	0.083	0.242	**0.917**	0.108	0.085	0.409	0.192	-0.092
8	0.051	0.391	0.235	0.237	-0.182	0.468	0.102	-0.044	0.193	0.846
9	0.356	0.348	**0.882**	0.278	-0.015	0.338	0.106	0.097	0.227	0.228
10	0.172	0.434	0.655	0.412	-0.017	0.299	0.097	0.025	0.188	0.146

High Risk subset (*n* = 151)

	1	2	3	4	5	6	7	8	9	10
1	**0.986**	0.203	0.365	0.230	0.253	0.075	0.219	0.269	0.270	0.271

2	0.260	**0.981**	0.453	0.515	0.180	0.346	0.117	0.109	0.277	0.157
3	0.381	0.635	0.477	**0.956**	0.279	0.472	0.238	0.217	0.313	0.222
4	0.275	0.226	0.224	0.263	**0.952**	0.163	0.198	0.189	0.301	0.397
5	0.325	0.347	0.262	0.328	0.338	0.151	0.283	0.302	**0.952**	0.450
6	0.282	0.096	0.189	0.174	0.162	-0.021	0.473	**0.956**	0.260	0.288
7	0.221	0.101	0.181	0.250	0.190	-0.015	**0.934**	0.467	0.296	0.191
8	0.098	0.292	0.171	0.348	0.097	**0.955**	-0.031	-0.046	0.149	0.144
9	0.433	0.324	**0.859**	0.264	0.125	0.164	0.157	0.159	0.180	0.180
10	0.230	0.427	0.832	0.407	0.117	0.201	0.137	0.133	0.138	0.184

Low Risk subset (*n* = 89)

	1	2	3	4	5	6	7	8	9	10
1	**0.955**	0.242	0.211	0.143	0.277	0.295	0.244	0.202	0.427	0.017
2	0.197	**0.962**	0.030	0.013	0.145	0.415	0.406	0.428	0.412	0.130
3	0.290	0.597	0.162	0.207	0.150	0.836	0.470	0.722	0.459	0.178
4	0.234	0.171	0.155	0.173	**0.909**	0.190	0.178	0.190	0.319	0.038
5	0.218	0.316	0.261	0.321	0.188	0.289	0.179	0.286	0.226	0.178
6	0.214	-0.010	**0.920**	0.463	0.203	0.140	0.098	0.041	0.218	-0.102
7	0.091	0.051	0.555	**0.893**	0.137	0.111	0.085	0.095	0.067	-0.107
8	0.118	0.313	-0.116	-0.118	0.029	0.350	0.194	0.395	0.273	0.827
9	0.276	0.387	0.140	0.007	0.288	0.281	0.469	0.296	0.760	0.200
10	0.149	0.398	0.127	0.088	0.215	0.331	**0.896**	0.361	0.446	0.064

Note. Rows of the table represent the first 10 factors extracted in the overall PCA solution (*N* = 282), with the first factor being R/S importance, the second factor being spirituality in nature, etc. (see text and Table 3). The columns represent the first 10 factors of the subgroup PCA solutions, as indicated by the sub-headers. The values in the inner portion of the table reflect Tucker's congruence coefficient φ (Lorenzo & ten Berge, 2006), with values in red representing "equality" (φ ≥ .95) and those in blue representing "fair similarity" (φ ≥ .85).
