# Supplementary material for: Understanding self-reported importance of religion/spirituality in a North American sample of individuals at risk for familial depression: A principal component analysis
Source: PLoS One. 2019 Oct 18;14(10):e0224141. doi: 10.1371/journal.pone.0224141 (PMC6799910; doi:10.1371/journal.pone.0224141)
Supplement: S3 Table — (PDF) [file pone.0224141.s003.pdf]

Table S3

S3 Table. Varimax-rotated principal component loadings for questionnaire items at most recent wave (Yr35).

	Factor							
	1	2	3	4	5	6	7	8
Items (*n* = 282 unless otherwise specified)	R/S Importance	Spirituality in Nature	Self-transcendence	Altruism	Love	Gratitude	Social Support	Mind Wandering
BEL_SALIENCE_2	**.844**							
BEL_SALIENCE_3	**.850**							
BEL_SALIENCE_1	**.830**							
INTRINS_REL_2	**.812**							
R/S IMPORTANCE	**.807**							
INTRINS_REL_3	**.788**							
INTRINS_REL_1	**.832**					.357		
REL_ENGAGE_1	**-.747**							
SELF_TRANS_16	**.805**		.402					
BEL_SALIENCE_5	**.598**							
REL_ATTENDANCE	**.541**							
REL_ENGAGE_2	**-.566**							
REL_ENGAGE_3	**-.597**		-.329					
FORGIVE_3	**.695**					.384	.358	
SELF_TRANS_17	**.760**	.323	.440				.335	
SELF_TRANS_4_Rev	**.629**						.371	
SELF_TRANS_14	**.693**		**.512**				.337	
SELF_TRANS_7	**.705**	.385	**.558**					
BEL_SALIENCE_4	.469							
SP_NATURE_3		**.822**	.365					
SP_NATURE_4		**.852**	**.528**					
ECO_AWARE_1		**.833**	.465					
SP_NATURE_1		**.771**	.372					

Table S3

SP_NATURE_6		**.796**	.478					
SP_NATURE_5		**.750**	.406					
SP_NATURE_2	.425	**.695**	.363					
SP_NATURE_7	.488	**.639**	.390				.313	
ECO_AWARE_6		**.694**	**.688**		.355			
ECO_AWARE_2		**.647**	**.519**					
SELF_TRANS_3		**.663**	**.703**					
SELF_TRANS_5		.326	**.729**					
SELF_TRANS_8	.422	.464	**.808**					
SELF_TRANS_10	.472	.393	**.729**				.398	
SELF_TRANS_18	.462	.377	**.735**		.326		.325	
SELF_TRANS_6	.464	.459	**.718**					
SELF_TRANS_2		.451	**.632**					
SELF_TRANS_25	.487	.319	**.653**					.334
SELF_TRANS_13	.458		**.604**					.322
SELF_TRANS_1			**.589**				.484	
SELF_TRANS_11			.448					
SELF_TRANS_15	.423	.407	**.553**					.351
ALTRUISM_1				**.763**				
ALTRUISM_2				**.763**				
ALTRUISM_4				**.741**				
ALTRUISM_3	.313			**.717**	.327			
ALTRUISM_6				**.589**				
ALTRUISM_5			.415	**.574**	.340	.328	.464	
PSYC_LOVE_2					**.793**			
ONT_LOVE_1			.334		**.784**			
ONT_LOVE_4			.315		**.727**	.313		
PSYC_LOVE_1					**.670**			
ONT_LOVE_2		**.503**	**.579**		**.726**			

Table S3

PSYC_LOVE_4					**.580**			
ONT_LOVE_3			.435		**.546**	.317	.311	
GRATITUDE_2						**.857**	.404	
GRATITUDE_1						**.806**	.370	
GRATITUDE_3						**.830**	.428	
GRATITUDE_4						**.772**	.351	
SOC_SUPPORT_3						.353	**.808**	
SOC_SUPPORT_1						.400	**.779**	
SOC_SUPPORT_2						.357	**.767**	
PSYC_LOVE_3						.337	**.535**	
SELF_TRANS_24								**.773**
SELF_TRANS_19								**.703**
SELF_TRANS_12								**.705**
SELF_TRANS_9			.448					**.685**
UNIVERSALITY_7	**.576**							
UNIVERSALITY_6	**.551**							
UNIVERSALITY_5	.480		.364					
COMPASSION_2				.378				
ECO_AWARE_4		.429						
REL_ENGAGE_4	**-.513**							
SELF_TRANS_23_Rev	.423							
BEL_SALIENCE_4	**.548**							
SELF_TRANS_21			.418					.447
SELF_TRANS_20		.448	.487					
UNIVERSALITY_8		.311	.358					
UNIVERSALITY_9	.410	.353	.460					
UNIVERSALITY_2		.338	.386					
UNIVERSALITY_1		.309						
UNIVERSALITY_3	.364	.364	.458					

Table S3

UNIVERSALITY_4		.380	.532		.357		
SOC_LOVE_3				.321	.307		
SOC_LOVE_1				.310	.302		
SOC_LOVE_2				.314		.360	
ECO_AWARE_5		.368					
ECO_AWARE_3		.319					
REL_ENGAGE_2	**-.566**						
SELF_TRANS_13	.458		**.604**				.322
SELF_TRANS_22			.374				.300
FORGIVE_1						.334	
FORGIVE_2						.337	
SOC_LOVE_4					.352		
SELF_TRANS_11			.448				
REL_COPING_1* (*n* = 139)	.455						
REL_COPING_2* (*n* = 139)	**.557**						
REL_COPING_3* (*n* = 139)	**.555**						
REL_COPING_4* (*n* = 139)	**.617**						
REL_COPING_5* (*n* = 139)	**.635**						
REL_COPING_6* (*n* = 138)	**.594**						
REL_SUPPORT_1* (*n* = 126)	.351						
REL_SUPPORT_2* (*n* = 126)	.335						

Correlations in **bold** are loadings > .5. Loadings < .3 are not listed.

* Variable/item not included in PCA due to missing data. Values listed are Pearson's correlations, which are in this case equivalent to factor loadings. Varimax (Vx) to Promax (Px) comparisons are color coded: GREEN: .3 ≤ Vx ≤ .5, Px ≥ .5; RED: Vx ≥ .5, Px ≤ .5; BLUE: Vx ≤ .3, Px ≥ .3 or ≥ .5 (**bolded**); *ITALICS*: not listed in Vx table.
